# Supplementary material for: Kinesin-2 and IFT-A act as a complex promoting nuclear localization of β-catenin during Wnt signalling
Source: Nat Commun. 2018 Dec 13;9:5304. doi: 10.1038/s41467-018-07605-z (PMC6294004; doi:10.1038/s41467-018-07605-z)
Supplement: Supplementary file 1 — Supplementary Information [file 41467_2018_7605_MOESM1_ESM.pdf]

Kinesin-2 and IFT-A act as a complex promoting nuclear localization of β-Catenin during Wnt signaling

Vuong et al

Supplementary Figure 1

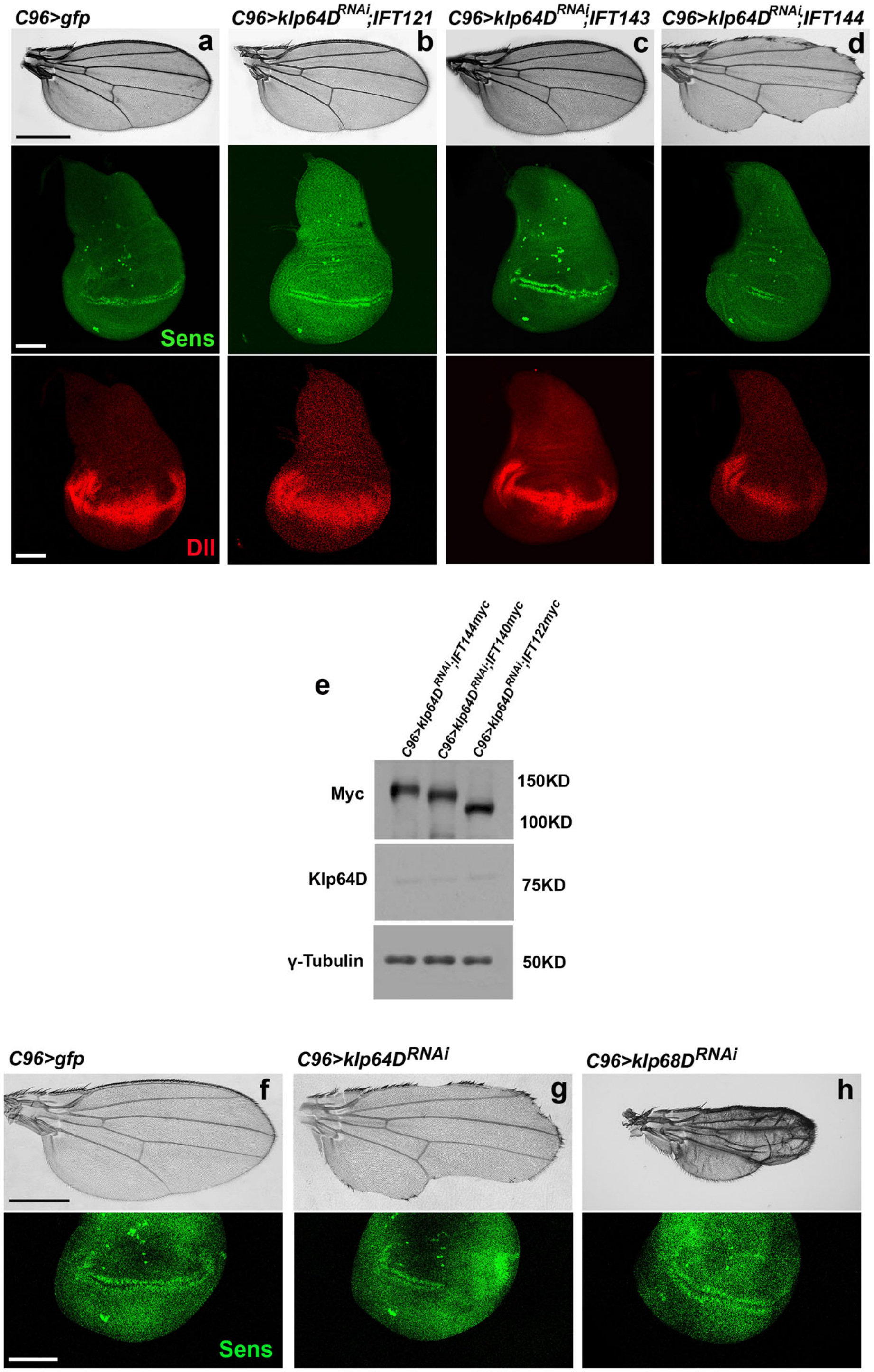

Supplementary Figure 1. Genetic interactions between kinesin-II, IFT-A components, and Axin.

(a) *C96-Gal4, UAS-GFP* (*C96>GFP*) control wing. C*96>GFP* (control) with normal Sens (in green) and Dll (in red) expression at/near the D/V boundary of wing imaginal discs.

(b-c) Notching/margin defects caused by *klp64D*^{RNAi} are suppressed by co-overexpression of IFT121 or IFT143, comparable to the other required IFT-A components. Reduced Sens and Dll expression are restored by co-overexpression of IFT-A components. Genotypes as indicated above panels, compare to genotypes in main Figure 1.

(d) The notching caused by *klp64D*^{RNAi} is not suppressed by overexpression of IFT144. The partial loss of Sens and reduction of Dll expression are not rescued by overexpression of IFT144. Note that IFT144 serves as a negative control, as it is not required for canonical Wnt/Wg β-catenin/Arm signaling.

(e) Levels of IFT-A components overexpression and Klp64D knockdown by *C96-Gal4*. Expression level of IFT-A components and Klp64D knockdown were checked by western blot using anti- Myc antibody and anti-Klp64D antibody. γ-Tubulin was used as loading control.

(f) *C96-Gal4, UAS-GFP* (*C96>GFP*) control wing. C*96>GFP* (control) with normal Sens (in green) expression at/near the D/V boundary of wing imaginal discs.

(g) *C96>klp64D*^{RNAi} shows wing notching phenotype and partial loss of Sens (in green).

(h) *C96>klp68D*^{RNAi} causes small wing phenotype and normal Sens expression at the D/V boundary of wing imaginal discs.

Scale bars represent 100μm, 30μm and 50μm. All tested wings show phenotype (n=100)

Supplementary Figure 2

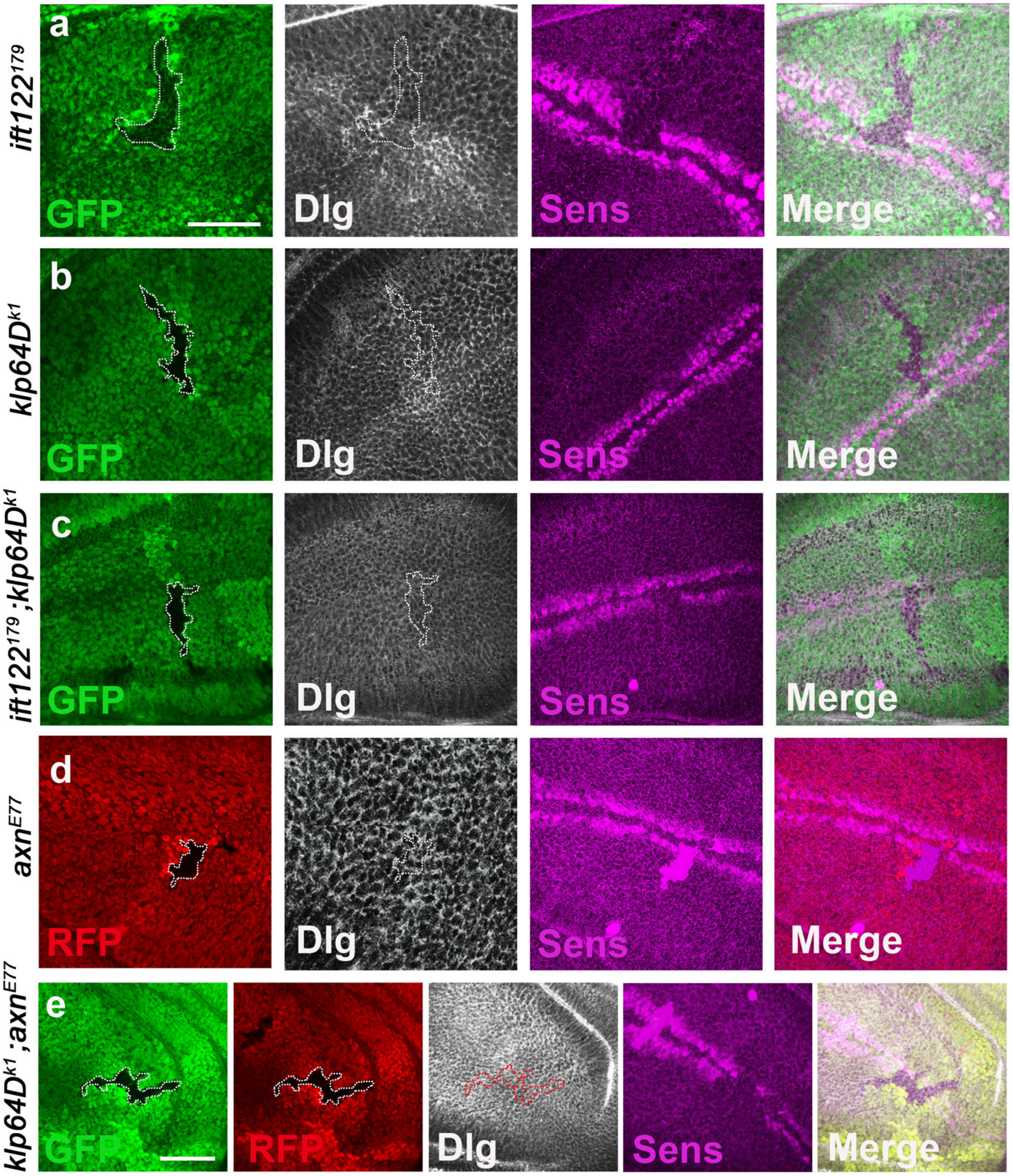

Supplementary Figure 2. The kinesin-II/IFT-A complex is required for Wg target gene expression. All panels show wing discs near the wing margin (evident by Sens expression, magenta), dorsal is up. Staining is GFP (green), RFP (red), Dlg (white), and Sens (magenta) as indicated. Genotypes are as labeled above panels. Dlg staining documents normal, wild-type cell architecture as control.

(a) Expression of Wg signaling target Sens is reduced in *ift122* mutant clones (n>20 clones in 10 different wing discs, marked by absence of GFP, outlined with white line). Junctional marker Dlg is expressed and localized normally in *ift122* mutant cells (in white); *ift122* mutant cells display loss or reduction of Sens expression (in magenta).

(b) Expression of Wg signaling target Sens is reduced in *klp64D* mutant clonal tissue (n>20 clones in 10 different wing discs, marked by absence of GFP, outlined with white line). Junctional marker Dlg is normally expressed and localized in *klp64D* mutant cells (in white), and Sens expression is lost (in magenta).

(c) Expression of Sens is lost in *ift122*, *klp64D* double mutant clones (n>20 clones in 10 different wing discs, marked by absence of GFP, outlined with white line). Junctional marker Dlg is normally expressed and localized in double mutant cells (in white), which display loss of Sens (in magenta).

(d) Ectopic expression of Wg signaling target Sens in *axin* mutant clones (n>20 clones in 10 different wing discs, marked by absence of RFP, outlined with white line). The cell junctional marker Dlg is normally expressed in *axin* mutant cells (in white), and mutant cells display ectopic Sens expression away from the margin (in magenta).

(e) Expression of Sens is lost in *klp64D*, *axin* double mutant clones (n>20 clones in 10 different wing discs, marked by absence of both GFP and RFP in (outlined with white line). Junctional marker Dlg is normally expressed and localized in both single and double mutant clones (in white), double mutant cells display loss of Sens expression (in magenta).

Scale bar represents 50 µm and 30 µm.

Supplementary Figure 3

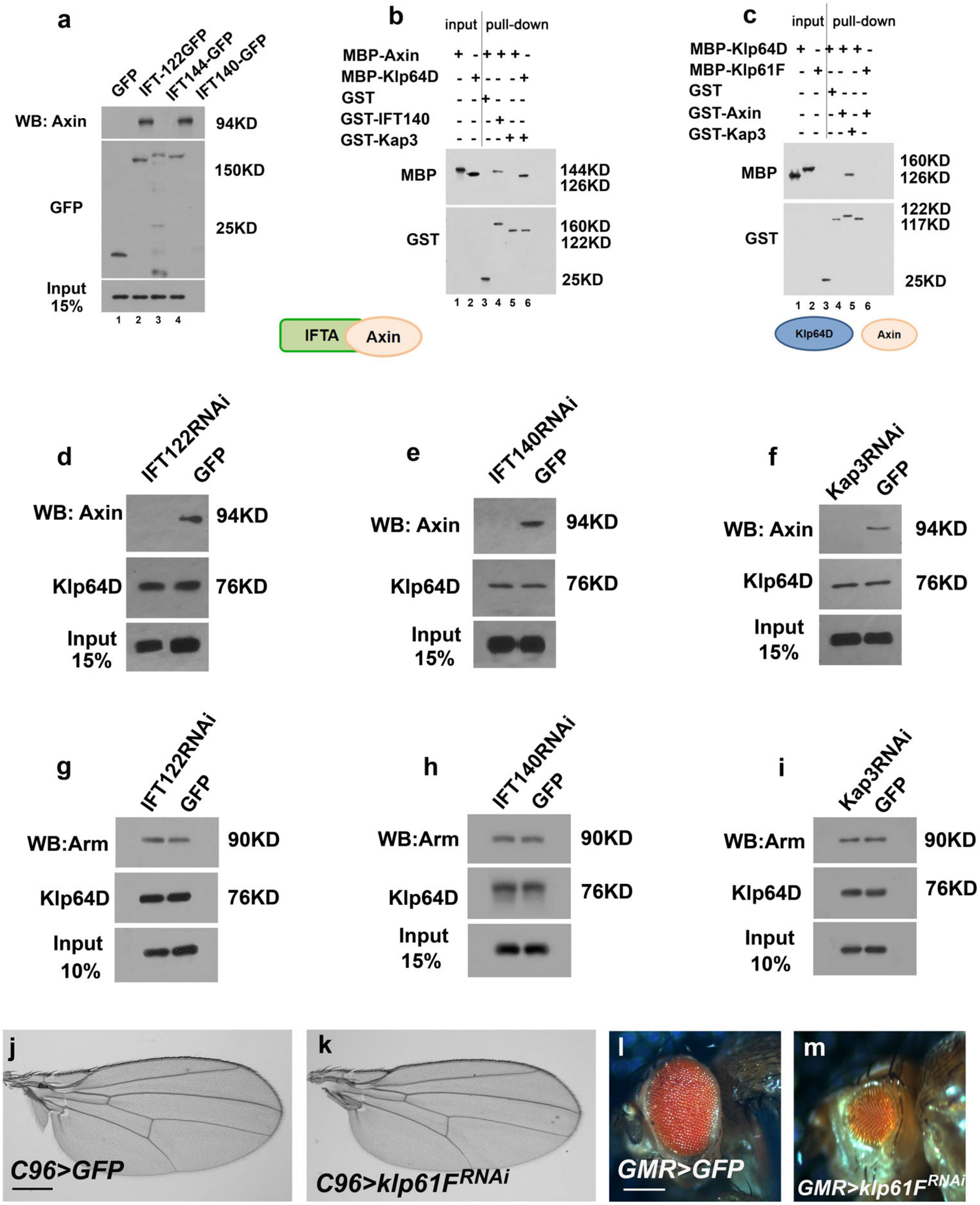

Supplementary Figure 3. Kinesin-II-IFTA complex and Axin are directly associated.

(a) Co-immunoprecipitation assay of Axin and IFTA from *tub>axinGFP* wing imaginal discs. Protein extracts from wing discs were immunoprecipitated with anti-GFP. Immune precipitates and input

(15% of wing disc lysates used in immunoprecipitation step) were analyzed by immunoblotting with antibody specific to Axin. Axin was co-immunoprecipitated by IFT-A components.

(b) Direct binding assay of IFT-A and Axin. Full length Axin (lane 1: input 10%) was pulled down by GST-IFT140 (lane 4), GST alone, negative control (lane 3), or GST- Kap3 (lane 5). Full length Klp64D (lane 2: 10% input) was pulled down by GST-Kap3 (lane 6) as positive control.

(c) Klp64D is not directly associated with Axin. Full length Klp64D (lane 1, 10% input) was not pulled down by GST (lane 3, control), GST-Axin (lane 4) was pulled down by GST-Kap3 (lane 5) as positive control. Full length Klp61F (lane 2: 10% input) was not pulled down by GST-Axin (lane 6, negative control).

(d-f) Co-immunoprecipitation assay of Klp64D and Axin from *nub>dcr2; >ift122*^{RNAi}; and *nub>dcr2; >ift140*^{RNAi}, or *nub>kap3*^{RNAi} and *nub>dcr2; >GFP* wing imaginal discs. Protein extracts from wing discs were immunoprecipitated with anti-Klp64D. The immune complexes and the input (15% of wing disc lysate used in the IP step) were analyzed by immunoblotting with antibody specific to Axin. (d-e) Klp64D does not form a complex with Axin-GFP in the absence of IFT122 or IFT140. (f) Klp64D also does not associate with Axin in the absence of Kap3.

(g-i) Co-immunoprecipitation assay of Arm and klp64D from *nub>dcr2; >ift122*^{RNAi}; and *nub>dcr2; >ift140*^{RNAi}, or *nub>kap3*^{RNAi} and *nub>dcr2; >GFP* wing imaginal discs. Protein extracts from wing discs were immunoprecipitated with anti-Klp64D. The immune complexes and the input (10%-15% of wing disc lysate used in the IP step) were analyzed by immunoblotting with antibody specific to Arm. (g-i) Klp64D forms a complex with Arm in the absence of IFT122 or IFT140 and Kap3.

(j) *C96-Gal4, UAS-GFP* (*C96>GFP*) control wing. (k) *C96>klp61F*^{RNAi} shows normal wing margin phenotype. (l) *GMR>GFP* shows normal eye. (m) *GMR-Gal4>klp61F*^{RNAi} shows small eye phenotype. Scale bars represent 50 µm. All tested wings and eyes show phenotype (n=100)

Supplementary Figure 4

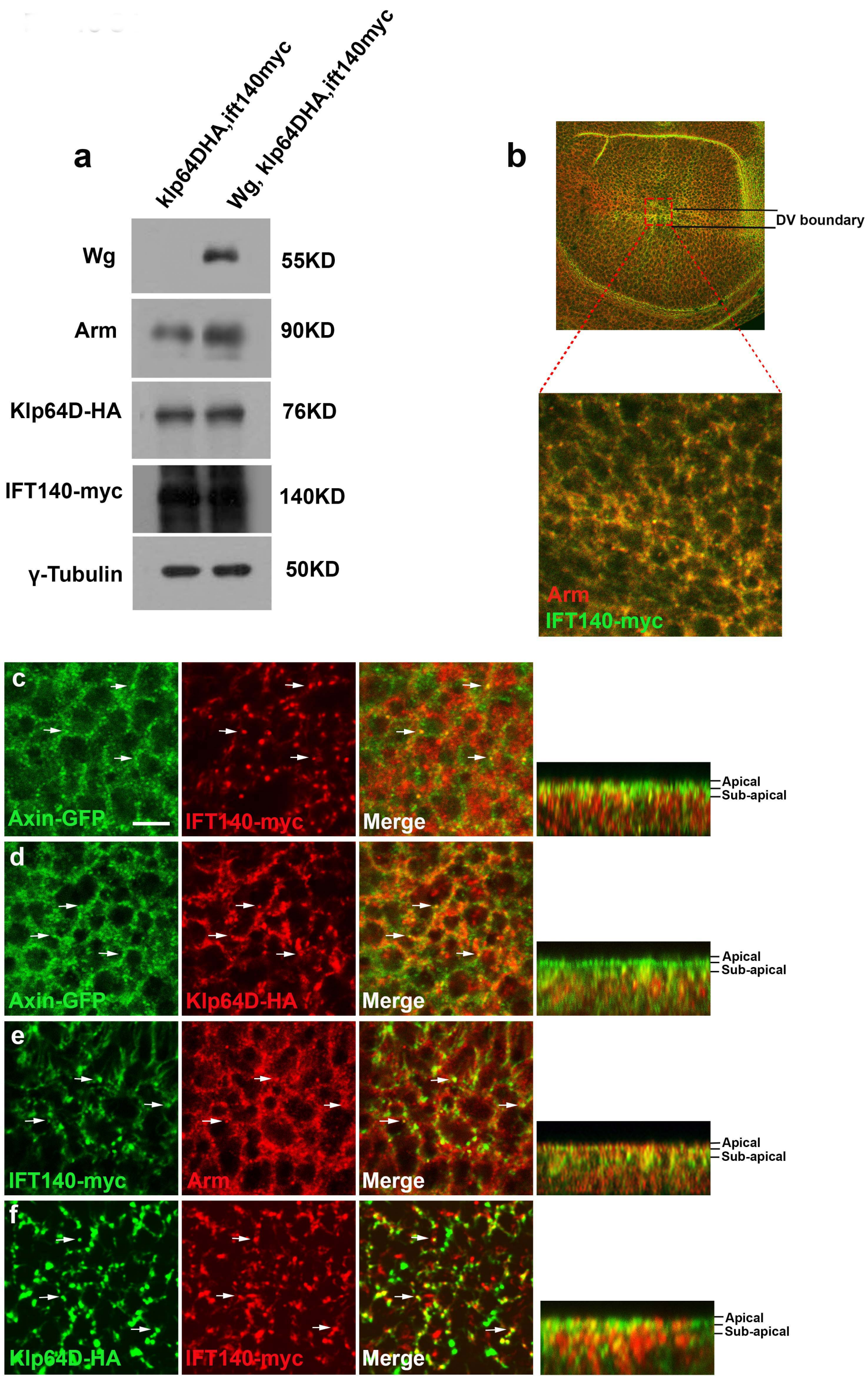

Supplementary Figure 4. Overlapping localization of Axin, Arm, and kinesin-II-IFTA complex. (a) Levels of Wg, Arm, Klp64D and IFT140 overexpression by *C805-Gal4*. Expression level of Wg, Arm, Klp64D and IFT140 were checked by western blot using anti-Wg, anti- Arm, anti-HA and anti- Myc antibody. γ-Tubulin was used as loading control.

(b) The magnification of sub- apical region at the D/V boundary of wing imaginal disc is shown in all panels c-f.

(c) Wing discs were stained for Axin-GFP (in green) and IFT140-myc (in red). Arrows indicate punctate staining of Axin-GFP overlapping with IFT140-myc. (d) Wing discs were stained for Axin-GFP (green) and Klp64D-HA (red). Arrows indicate punctate staining of Klp64D-HA overlapping with Axin-GFP. (e) Wing discs were stained for IFT140-myc (green), and Arm (red). Arrows indicate punctate staining of IFT140-myc overlapping with Arm. (f) Wing discs were stained for Klp64D-HA (green), and IFT140-myc (red). Arrows indicate punctate staining overlapping between Klp64D-HA and IFT140-myc. Scale bar represents 30µm.

Supplementary Figure 5

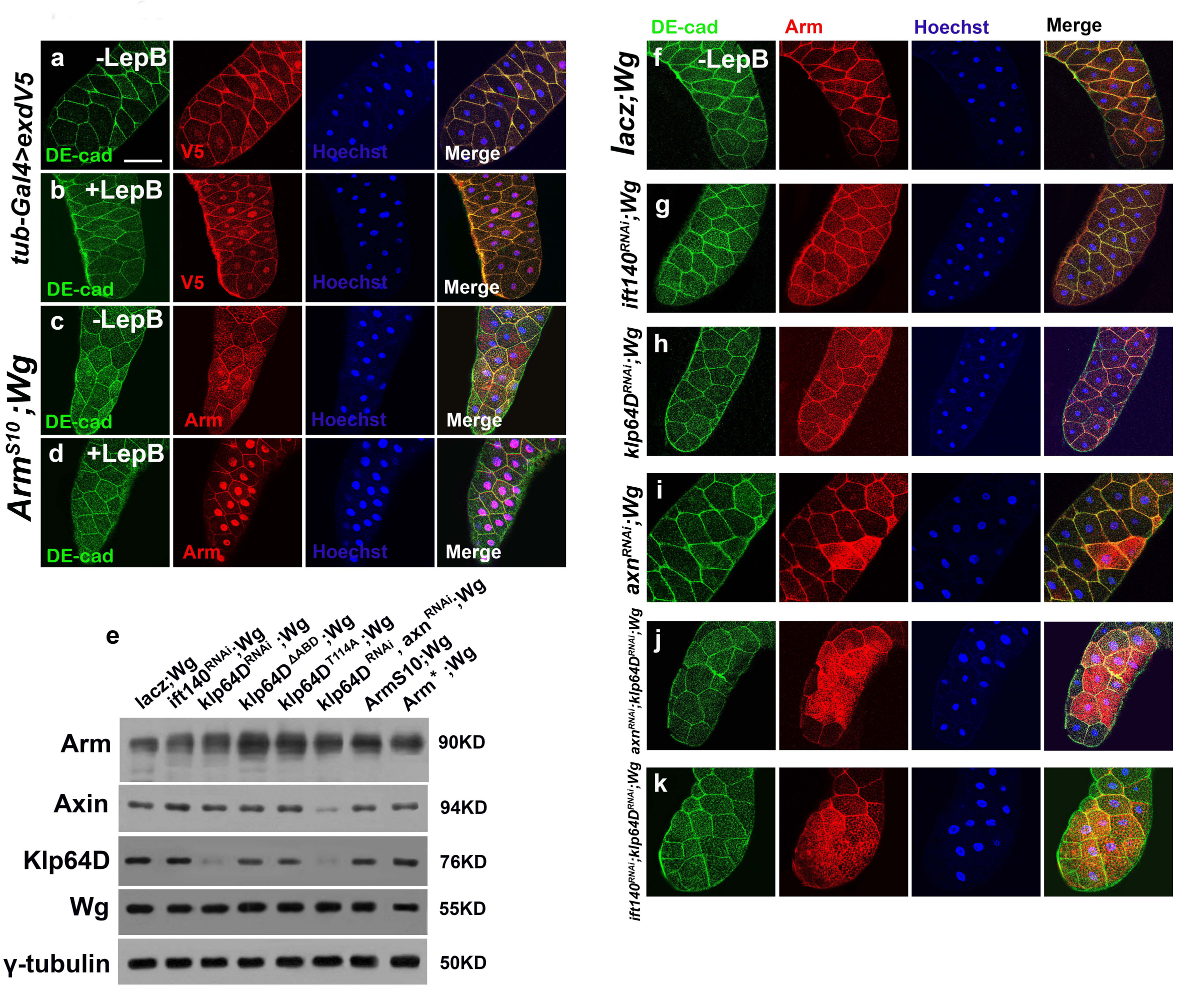

Supplementary Figure 5. Arm levels in cytoplasm and nucleus in the absence of kinesin-II/IFT-A complex without LepB treatment.

Salivary glands were stained with DE-cad (green, control and membrane marker), Arm (red), and Hoechst (blue). (a) Only few cells show detectable nuclear localization of ExdV5 without LepB treatment. (b) ExdV5 is involved in nuclear shuttling and remain inside the nucleus in LepB treated salivary glands.

(c) *C805>ArmS10 >Wg*: note that only very few cells show detectable nuclear localization of ArmS10 without LepB treatment. (d) *C805>ArmS10>Wg* with LepB treatment: note that Arm is under these conditions mainly localized in the nucleus and overlaps with nuclear markers (Hoechst, blue).

(e) Western blot to test expression levels of Arm, Klp64D, Axin and Wg in

C805>lacz>Wg;

C805>ift140^{RNAi}>Wg;

C805>klp64D^{RNAi}>Wg;

C805>klp64D$^{\Delta ABD}$>Wg;

C805>klp64D^{T114A}>Wg;

C805>klp64D^{RNAi}>axin^{RNAi}>Wg;

C805>ArmS10>Wg

and *C805>Arm*>Wg*

with the treatment of Lep B. Note that loss of Axin protein and reduced Klp64D protein levels, confirming that RNAi constructs are working.

All f-k panels are without LepB treatment and with Wg expression from the salivary gland specific driver, compare to main Figure 5. Scale bar represents 50 µm.

(f) *C96>lacz>Wg*: Arm is mainly detected in the cytoplasm and at the cell membrane.

(g-h) Reduced levels of IFT140 (g) and Klp64D (h) reveal Arm expression levels are reduced in some cells and part of the membrane.

(i) Axin knock-down leads to abnormal high Arm levels in the cytoplasm, but no detectable nuclear Arm staining without the drug treatment.

(j) Cytoplasmic Arm levels are high when both Axin and Klp64D are knocked down.

(k) Cytoplasmic Arm levels remain high in double knock-down backgrounds of Axin and IFT140.

Supplementary Figure 6

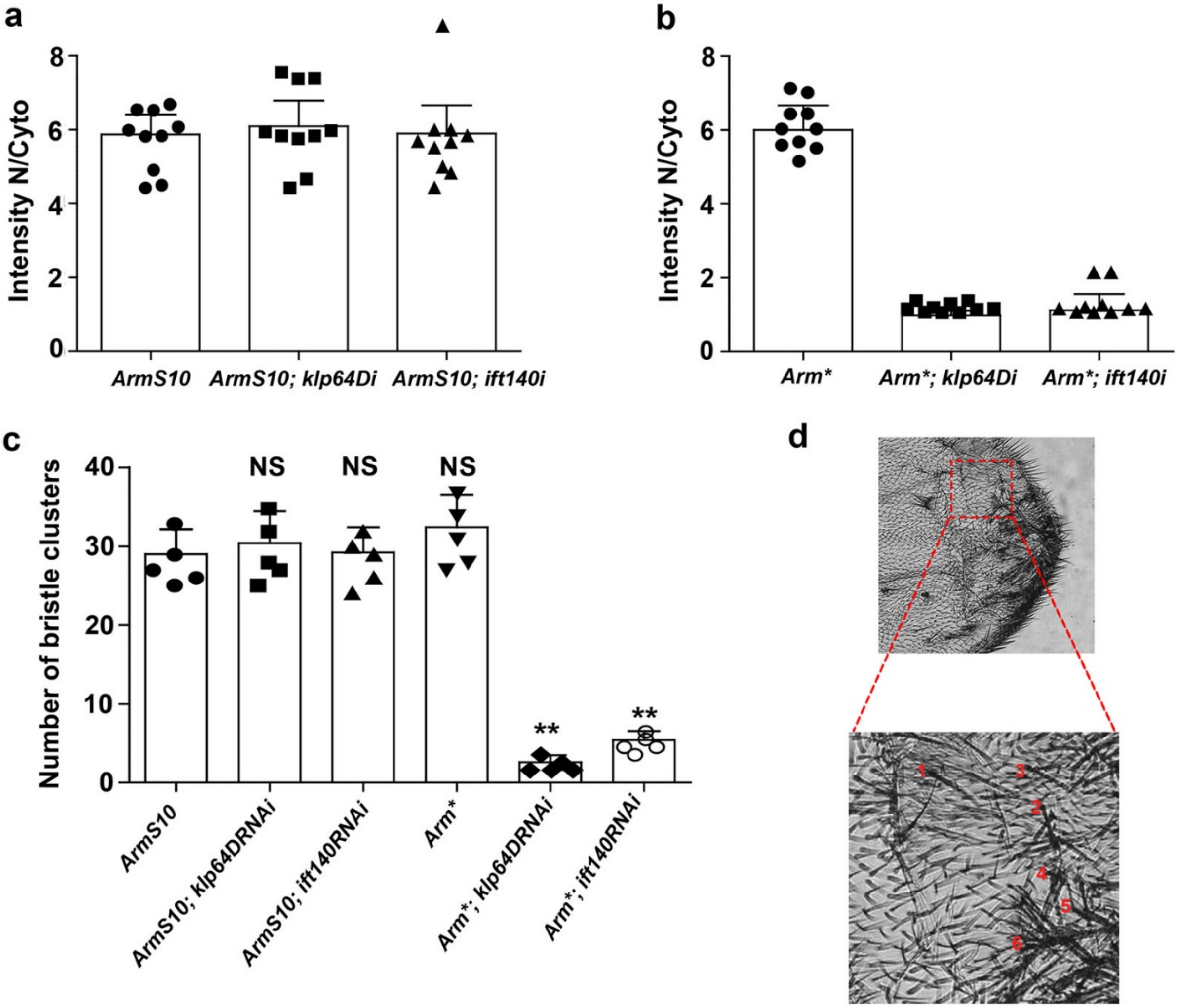

Figure S6. Quantification of nuclear Arm/β-catenin signal and the number of ectopic bristles in Fig.6

(a) Quantification of nuclear β-catenin IF signal in *C850-Gal4>ArmS10, C850-Gal4>ArmS10; klp64D RNAi and C850-Gal4>ArmS10; ift140RNAi*. (b) Quantification of nuclear β-catenin IF signal in *C850-Gal4>Arm**, *C850-Gal4> Arm***; klp64D RNAi and C850-Gal4> Arm***; ift140RNAi*. Y axis denominates the ratio of gray intensity values of selected regions (area of 4µm x 4µm) within nuclei and cytoplasm of individual cells (membrane-associated β-catenin was purposely excluded). Mean $\pm$ s.d. of values obtained in randomly selected cells are shown from three independent experiments; Student's t test: *** indicate p<0.001.

(c) Quantification of the number of ectopic bristles per wing area. Bars represent mean +/- SD, n=10 wings per genotype, $^{**}p<0.001$; *NS* = non-significant (Student's t test).

(d) The number of ectopic bristle clusters per wing area.

Supplementary Figure 7

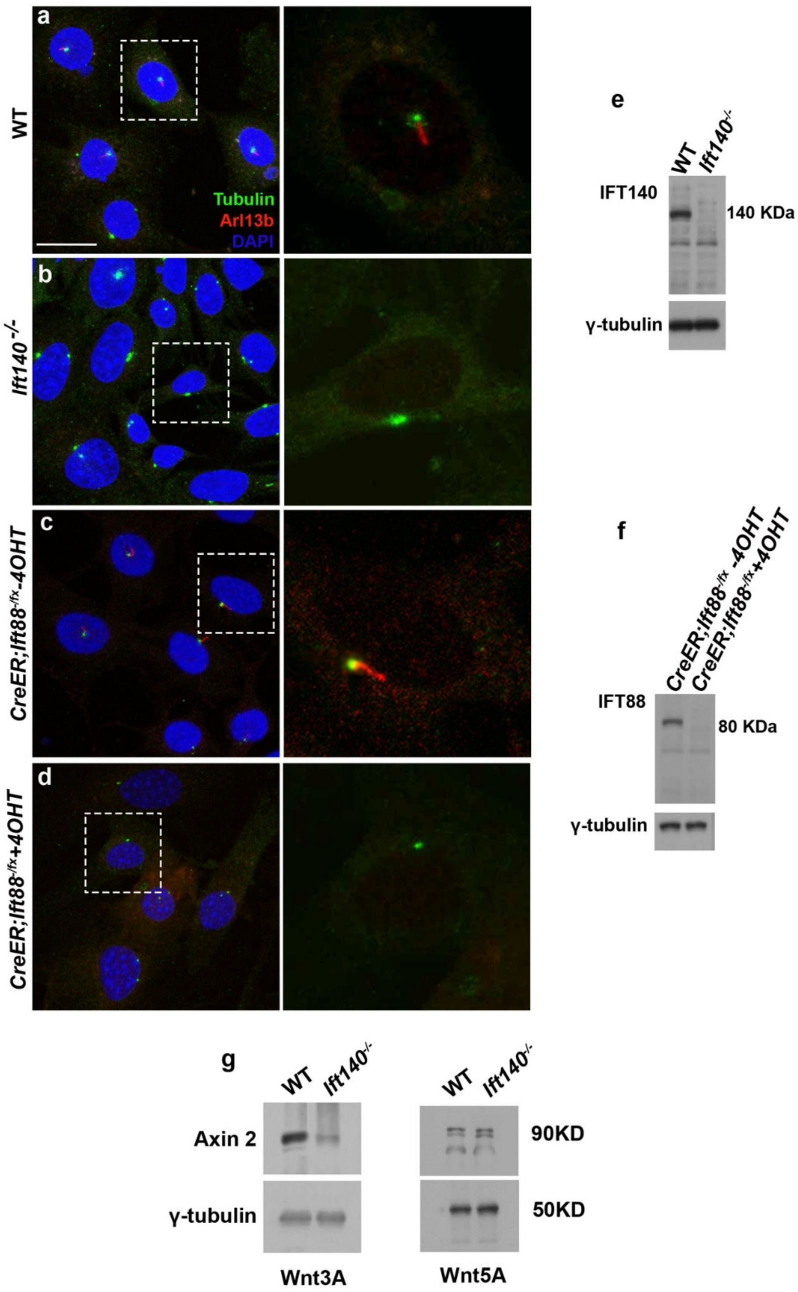

Figure S7. Primary cilia structure in *IFT140* and *IFT88* MEFs and Axin2 expression levels in the MEFs. Confocal images of immunofluorescence (IF) of γ-tubulin (green), Arl13b (red) and nuclei (blue) in MEF cells. Scale bar represents 100 µm.

(a) Wild-type MEFs show normal primary cilia structure with the basal body (in green), cilia (in red) and nuclei (in blue).

(b) No cilia are detected in the *Ift140*$^{-/-}$ MEFs.

(c-d) The cilia structure is normal in the absence of tamoxifen inducible *CreER; Ift88*$^{-/fx}$ MEFs cultured (c) but remained undetectable cilia in the presence of 50 nM 4-hydroxytamoxifen (4OHT) induced *CreER; Ift88*$^{-/fx}$ MEFs (d).

(e-f) Western blots monitoring the expression/knock out of IFT140 and IFT88 in control and experimental MEFs in total cell extracts. IFT140 and IFT88 were present in cell extracts of control MEFs but undetectable in *Ift140*$^{-/-}$ MEFs (e) and in tamoxifen induced *CreER; Ift88*$^{-/fx}$ MEFs (f).

(g) Western blots show the expression of Axin2 in IFT140 control and *Ift140*$^{-/-}$ MEFs in total cell extracts. Axin2 level expression is reduced in *Ift140*$^{-/-}$ MEFs treated with Wnt3a media comparing to the wild-type MEFs. However, the levels of Axin2 expression are the same in both *Ift140*$^{-/-}$ MEFs and wild-type MEFs treated with Wnt5a media.

Scale bars represent 100µm, 30µm and 50µm.

Supplementary Figure 8. Original gels of all western blots in the paper.

Figure 3a

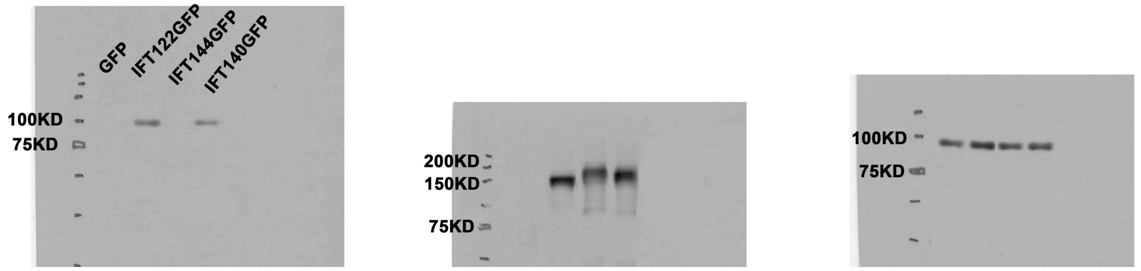

Figure 3c

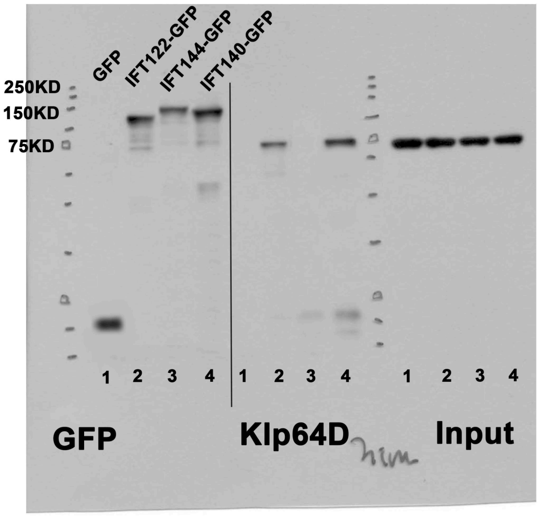

Figure 3d

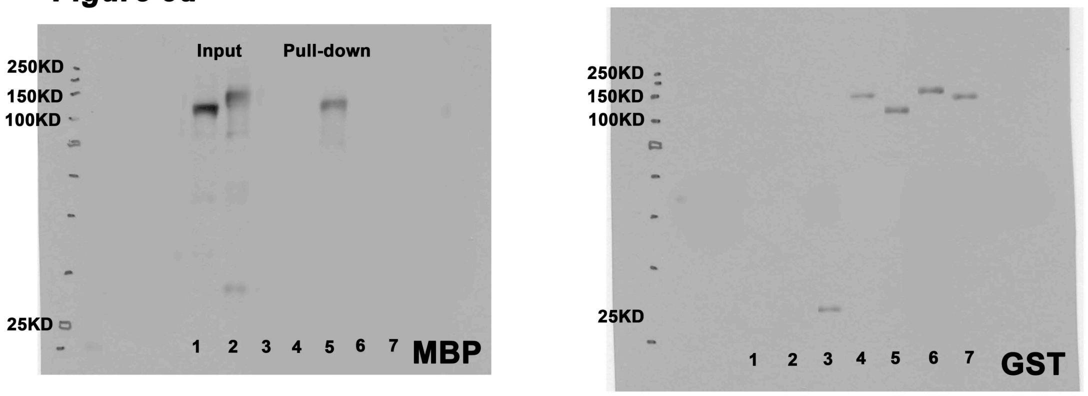

Figure 3e

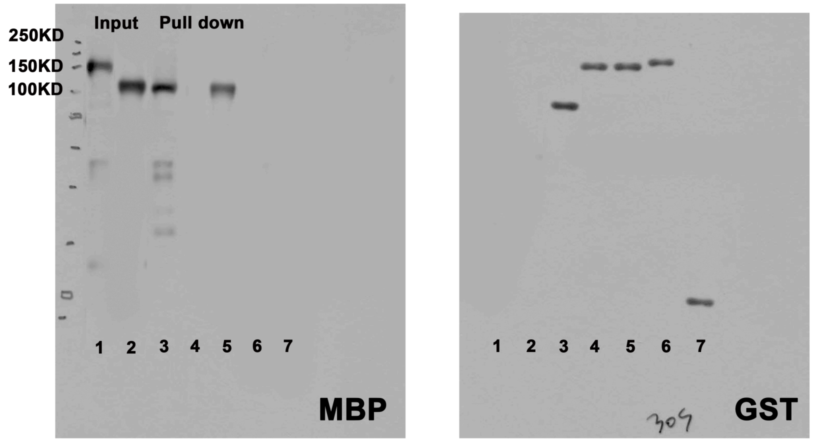

Figure 7a

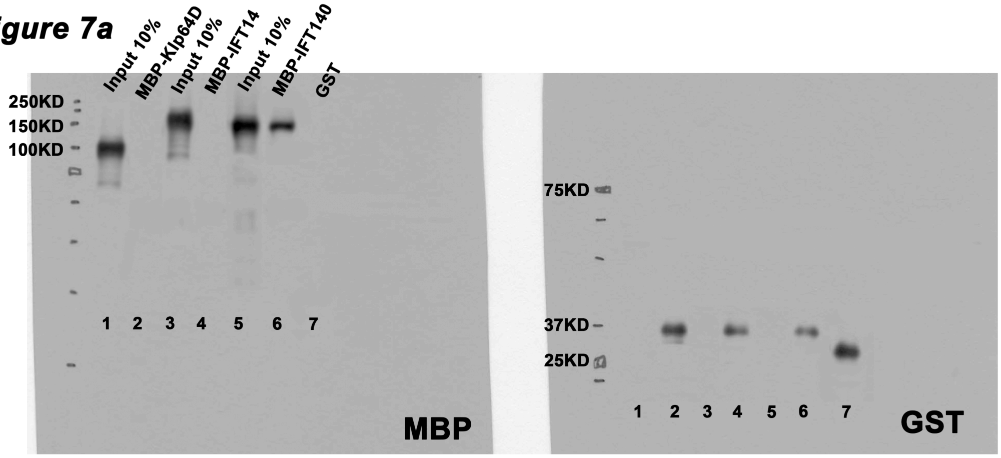

Figure 8j

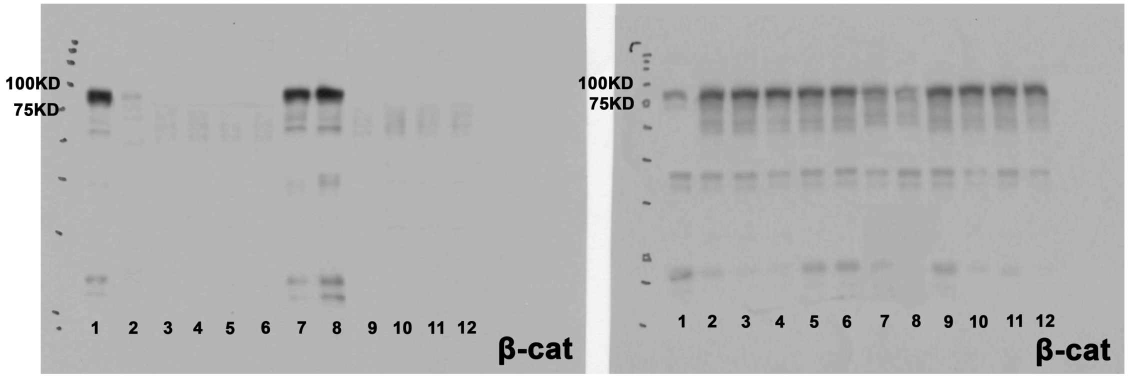

(Nuclear fraction) **(Cytoplasmic fraction)**

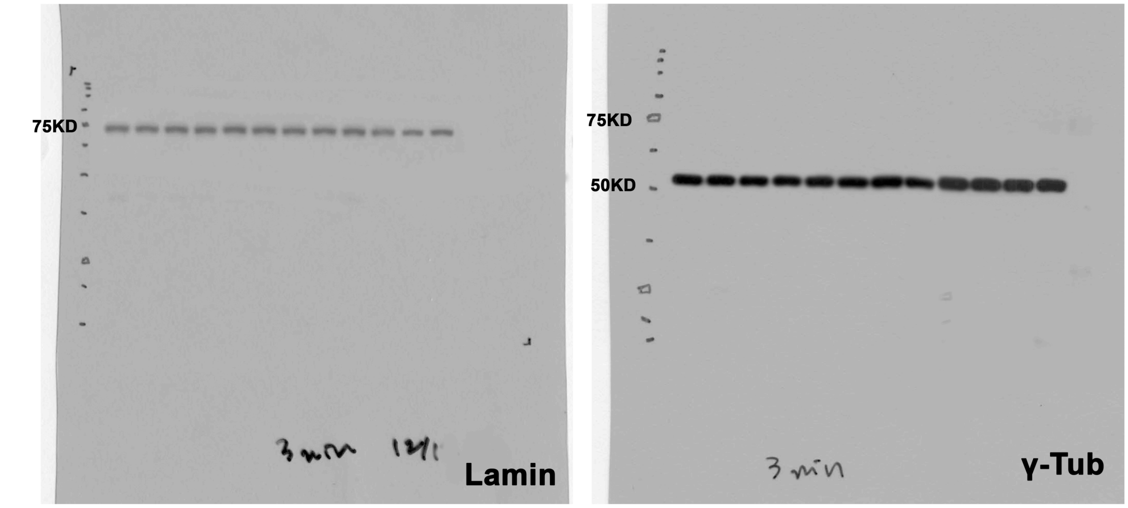

Figure 9a

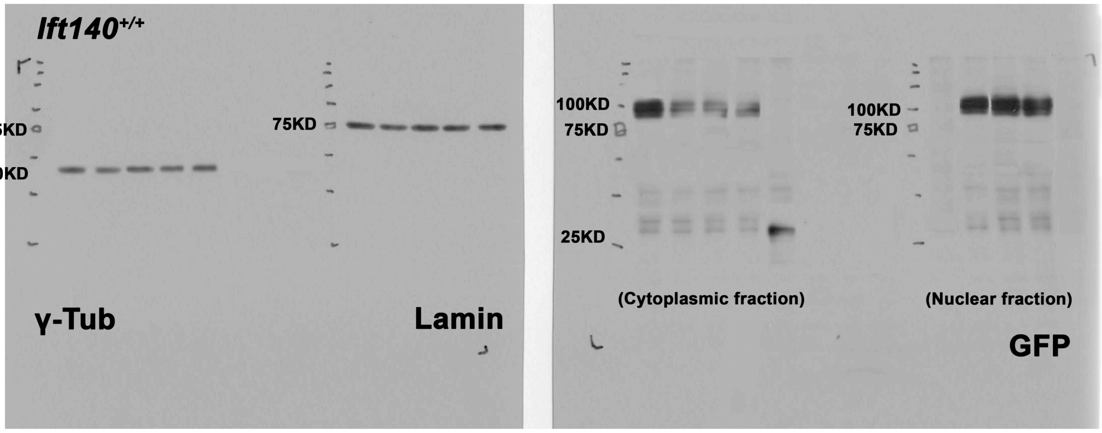

Figure 9b

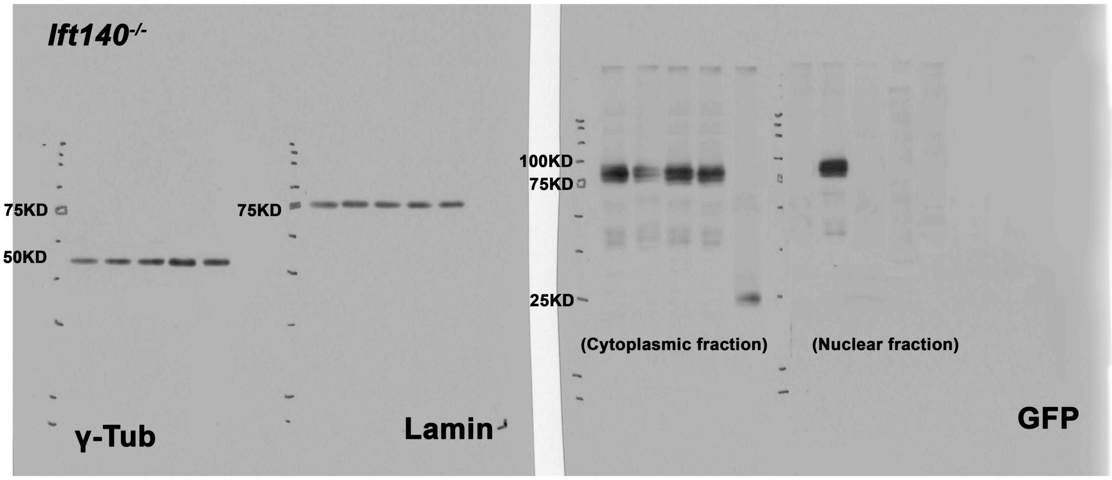

Figure 9c

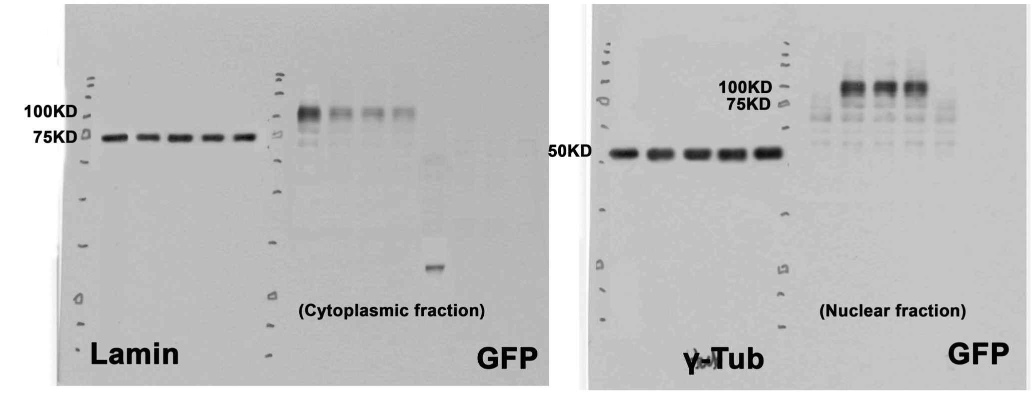

Figure 9d

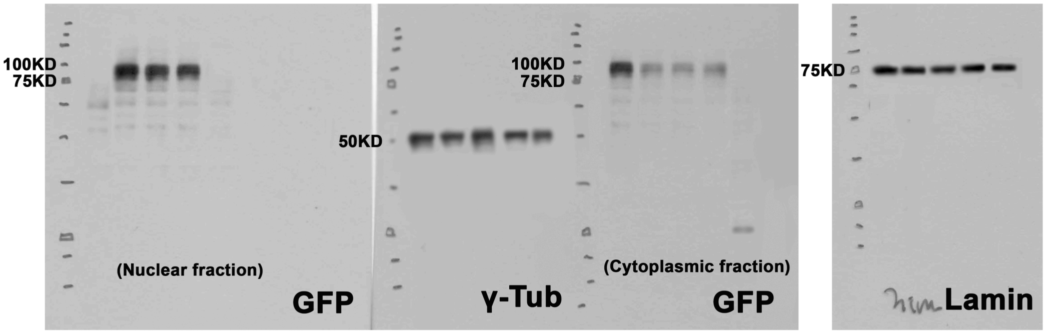

FigS3c

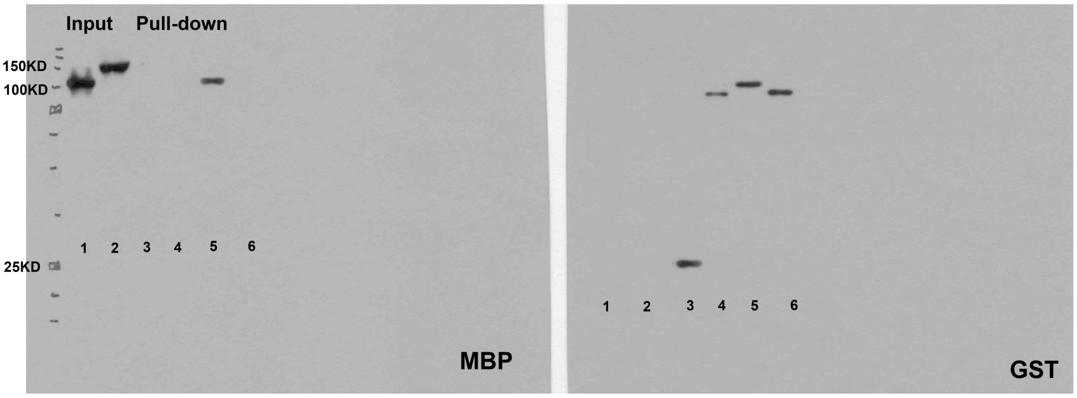

FigS3d

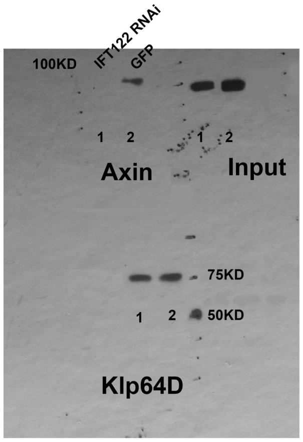

FigS3e

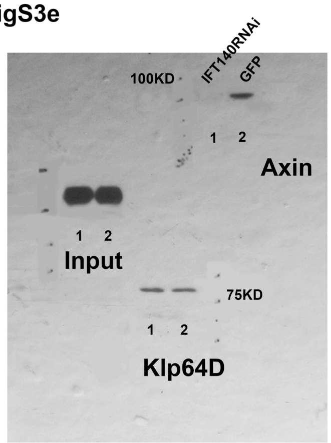

FigS3f

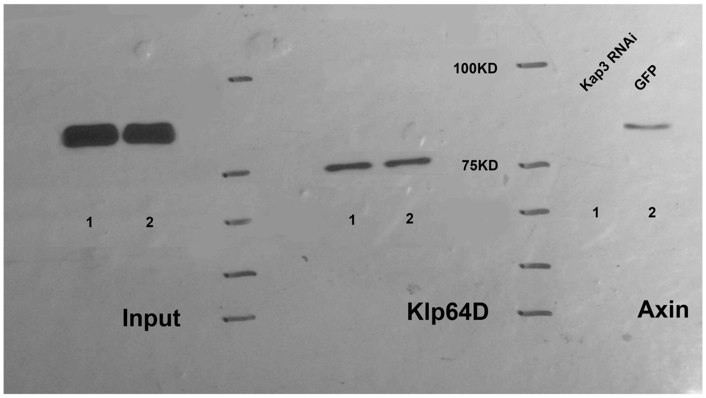

Figure S3g

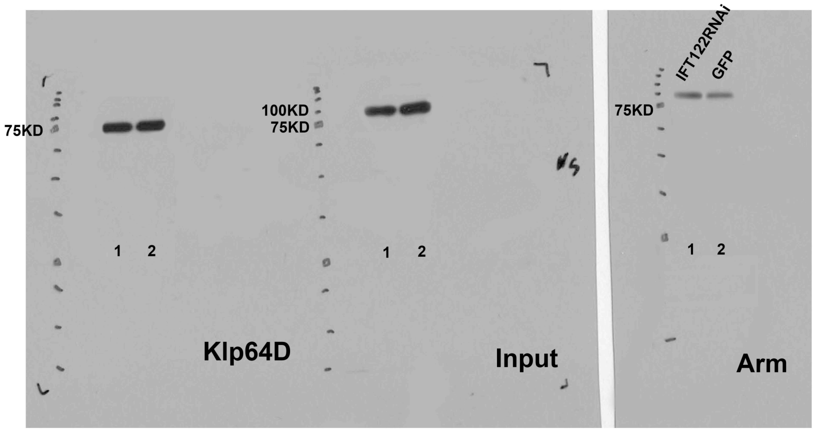

Figure S3h

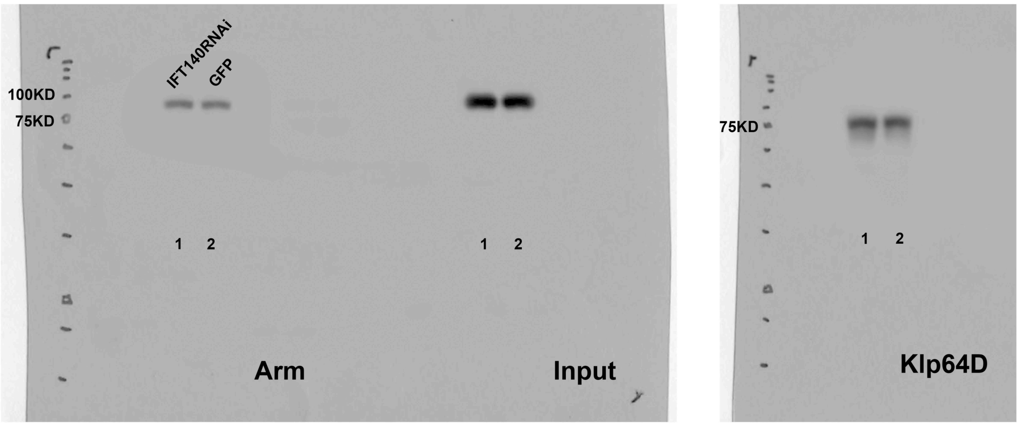

Figure S3i

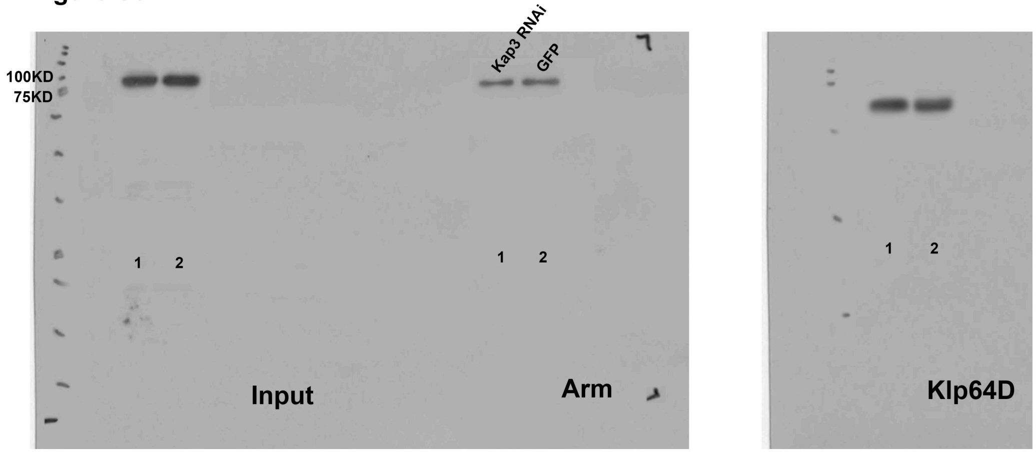

Figure S4a

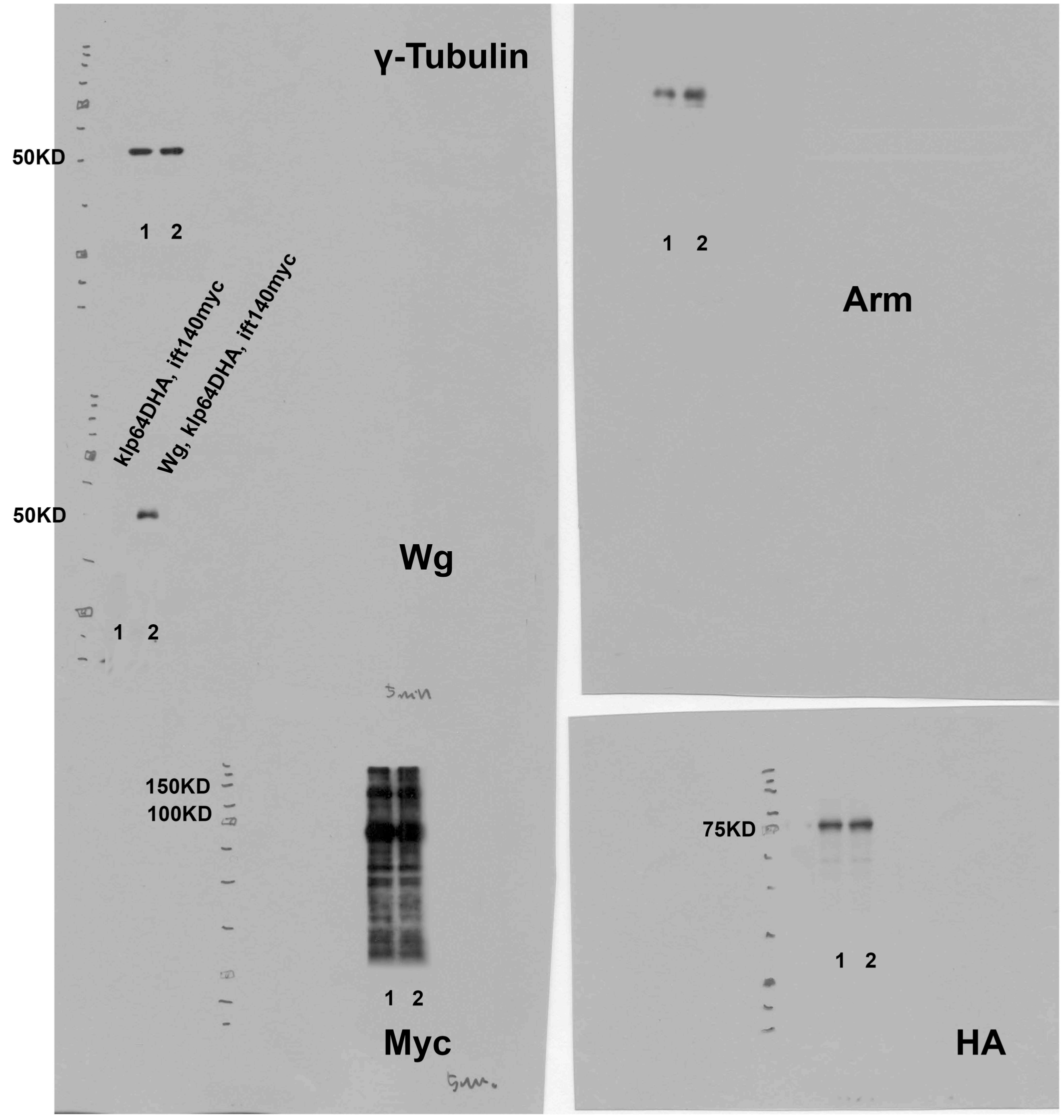
γ-Tubulin
50KD
1 2
klp64DHA, ift140myc
Wg, klp64DHA, ift140myc
50KD
Wg
1 2
Arm
1 2
150KD
100KD
1 2
Myc
75KD
1 2
HA

Figure S5

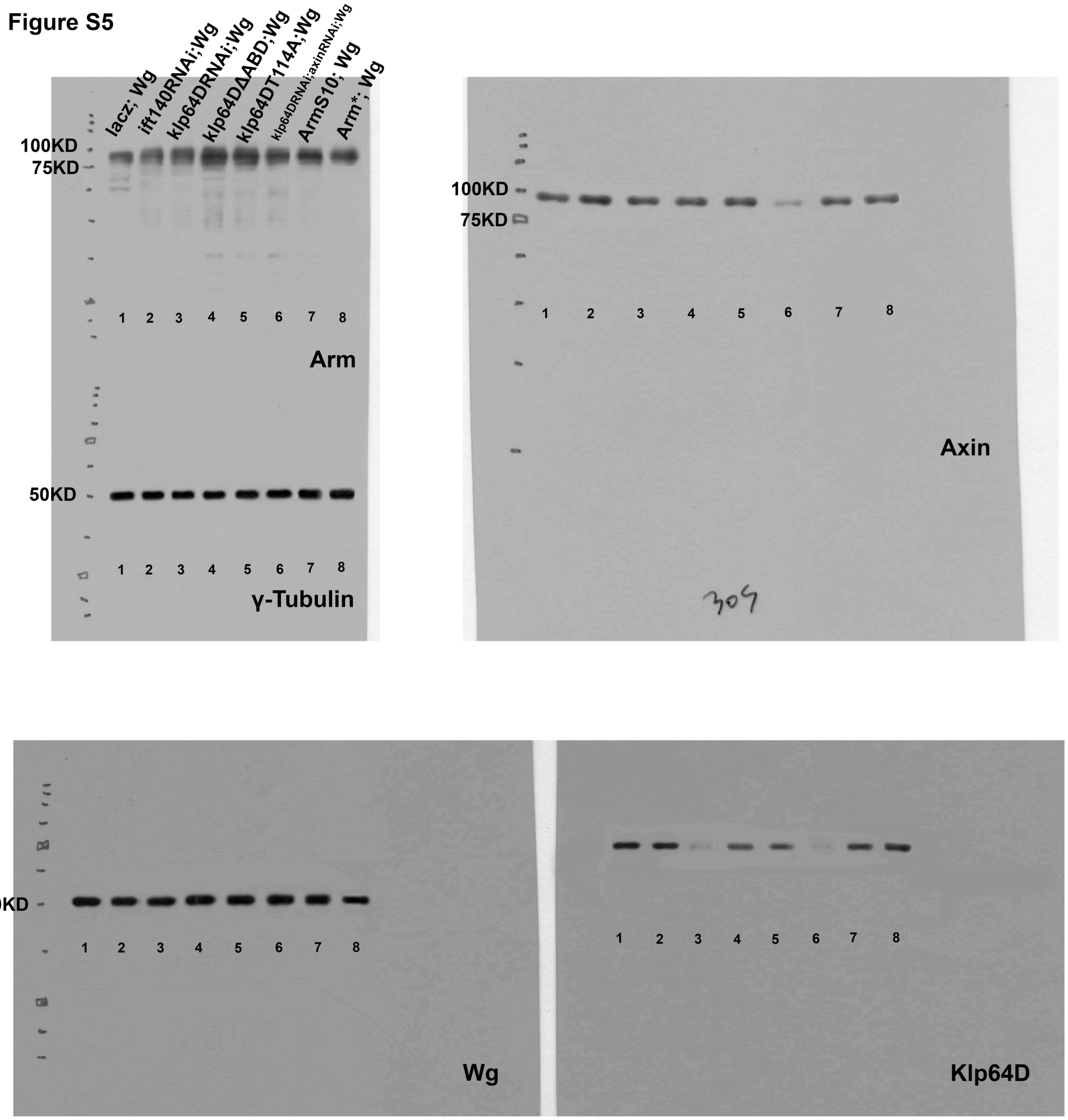
lacz; Wg
ift140RNAi;Wg
klp64DRNAi;Wg
klp64DΔABD;Wg
klp64DT114A;Wg
klp64DRNAi;axinRNAi;Wg
ArmS10; Wg
Arm*; Wg
100KD
75KD
1 2 3 4 5 6 7 8
Arm
50KD
1 2 3 4 5 6 7 8
γ-Tubulin
100KD
75KD
1 2 3 4 5 6 7 8
Axin
50KD
1 2 3 4 5 6 7 8
Wg
1 2 3 4 5 6 7 8
Klp64D
